# Targeting Epigenetic Alterations Linked to Cancer-Associated Fibroblast Phenotypes in Lung Cancer

**DOI:** 10.3390/cancers16233976

**Published:** 2024-11-27

**Authors:** Kostas A. Papavassiliou, Amalia A. Sofianidi, Vassiliki A. Gogou, Athanasios G. Papavassiliou

**Affiliations:** 1 First University Department of Respiratory Medicine, ‘Sotiria’ Chest Hospital, Medical School, National and Kapodistrian University of Athens, 11527 Athens, Greece; konpapav@med.uoa.gr (K.A.P.); gogvanessa@gmail.com (V.A.G.); 2 Department of Biological Chemistry, Medical School, National and Kapodistrian University of Athens, 11527 Athens, Greece; amsof.00@gmail.com

The focus in cancer research and treatment has recently shifted from being primarily tumor-centric to emphasizing the tumor microenvironment (TME) [1]. The TME is a dynamic ecosystem consisting of malignant cells, immune cells, stromal cells, blood and lymph vessels, neuron fibers, extracellular matrix (ECM), and various acellular components [1]. Among these, fibroblasts that thrive within the TME, now referred to as cancer-associated fibroblasts (CAFs), have emerged as key players in tumor biology [2]. Indeed, CAFs represent a highly heterogeneous cell population, varying not only in their origins and molecular phenotypes [3] but also in their distinct epigenetic profiles [4]. Recently, epigenetic alterations have gained recognition as pivotal modulators of CAF activation and heterogeneity [4]. Remarkably, in 2022, Hanahan introduced the updated hallmarks of cancer, underscoring the critical role of epigenetic changes in acquiring hallmark capabilities during tumor development and progression [5].

While genetic alterations have been identified in CAFs, this type of fibroblast is generally more genetically stable compared to tumor cells [6]. However, epigenetic modifications play a significant role in shaping the tumor-promoting properties of CAFs. CAFs are influenced by three main types of epigenetic regulatory mechanisms: DNA methylation, histone modification (i.e., post-translational covalent modifications to histone tails that affect the structural state of chromatin, hence the transcriptional status of genes within particular locations), and non-coding RNAs (ncRNAs) [7]. More specifically, global hypomethylation, alongside targeted hypermethylation of key transcription factors like SMAD3, appears to be a characteristic of lung cancer CAFs [7]. Additionally, N-methyltransferases, metabolic enzymes regulating cell metabolism and triggering epigenetic modifications, are overexpressed in various tumors and are associated with poor prognosis in non-small-cell lung cancer (NSCLC) patients [7,8]. When it comes to ncRNA alterations in CAFs, diverse microRNAs (miRNAs) have been identified as being either upregulated or downregulated in lung cancer, driving CAFs towards a tumor-promoting phenotype [7]. Notably, cytokines like transforming growth factor beta (TGF-β) have proven to be key drivers of the epigenetic changes mentioned above [7].

Rather than concentrating solely on discovering new treatment strategies targeting DNA mutations, therapies aimed at addressing epigenetic mutations are particularly promising due to the potentially reversible nature of these mutations [9]. Deregulated histone deacetylases (HDACs)—enzymes that remove an acetyl group from histone lysine residues, generally leading to transcriptional inactivation of the involved DNA attributable to chromatin condensation—have been strongly implicated in abnormal gene silencing and tumorigenesis, and the antitumor potential of HDAC inhibitors has been well recognized for years [10]. Given the aberrant involvement of HDACs in NSCLC progression [11], targeting these enzymes presents a promising therapeutic strategy against CAFs in solid tumors. Notably, the HDAC inhibitor scriptaid (6-(1,3-dioxo-1H-benzo[*de*]isoquinolin-2-yl)*N*-hydroxyhexanamide) has shown preclinical therapeutic effects against activated CAFs in melanoma and breast cancer [12]. Although scriptaid has yet to enter clinical trials, its derivative, suberoylanilide hydroxamic acid (SAHA), also known as Vorinostat, has been tested in clinical trials for NSCLC in combination with other chemotherapeutic agents [13] and the programmed cell death protein 1 (PD-1) inhibitor pembrolizumab [14]. These studies have highlighted improved therapeutic efficacy and increased patient survival when Vorinostat is incorporated into combination regimens. In similar terms, fimepinostat (CUDC-907), an orally available small-molecule dual inhibitor of phosphoinositide 3-kinase (PI3K) class I isoforms and HDAC, has demonstrated the ability to suppress cancer cell proliferation, reduce collagen production and ECM deposition, and restrain the migration and invasion capabilities of CAFs in vivo in NSCLC models [15]. Currently, clinical trials are also underway to assess the efficacy of HDAC inhibitors in action. NCT01928576 is a phase-II study of epigenetic therapy including azacytidine (a cytotoxic nucleoside analog) and entinostat, a synthetic benzamide-derivative HDAC inhibitor, with simultaneous administration of the PD-1 inhibitor nivolumab in patients with metastatic NSCLC. Another phase-II trial, NCT05141357, evaluates the orally bioavailable class-I selective HDAC inhibitor tucidinostat (HBI-8000) in combination with nivolumab for advanced or metastatic NSCLC.

Regarding miRNAs, a CAF-specific long ncRNA (lncRNA), LINC01614, enhances the glutamine uptake of CAFs in the TME and is associated with poor prognosis in lung cancer patients [16]. The deletion of LINC01614 significantly reduced the metastatic potential of lung cancer in vivo, highlighting new opportunities for targeting miRNAs as part of epigenetic therapy [16]. Furthermore, given the prominent role of SMAD3 in shaping the epigenetic profile of lung cancer CAFs, the SMAD3 inhibitor SIS3 (a pyrrolopyridine that selectively hinders TGF-β1-dependent SMAD3 phosphorylation) has been employed in preclinical models, demonstrating favorable therapeutic effects in lung cancer [17].

Since TGF-β is implicated in molding the epigenetic landscape of CAFs, efforts could be oriented towards targeting this paracrine signaling factor. Galunisertib (LY2157299) is a small-molecule antagonist of TGF-β receptor 1 (TGFβR1) [18]. It has been tested in phase-I and -II trials enrolling NSCLC patients with positive results; when galunisertib was administered in combination with nivolumab, no patients were found to have antibodies against nivolumab, which is promising for the optimal use of immunotherapy in NSCLC [19]. Moreover, Shi et al. revealed that LY2109761, an orally active, potent TGFβR1 inhibitor, decreased the expansion of squamous cell carcinoma (SCC)-associated CAFs in vivo [20]. Lastly, inhibition of autophagy with the use of hydroxychloroquine abrogated CAF activation and TGF-β production, impeding lung adenocarcinoma progression [21].

In conclusion, Stephen Paget’s enduring “seed and soil” theory of cancer remains profoundly relevant, emphasizing the crucial role of the TME, including CAFs, in tumor development and progression [22]. To effectively combat cancer and abate its associated mortality, we must innovate and refine our therapeutic strategies. Targeting the reversible epigenetic landscape of CAFs represents a hopeful approach in this ongoing fight. Intriguingly, the epigenetic profile of CAFs holds even greater potential value; Luo et al. recently demonstrated that the expression of the *leucine rich repeat containing 3B* (*LRRC3B*) gene, a tumor suppressor gene engaged in the antitumor immune microenvironment, and the associated DNA methylation at the *LRRC3B* promoter region may serve as a valuable predictive biomarker for anti-PD-1 therapy in NSCLC patients [23]. May the years ahead of investigation elucidate the transformative potential of targeting epigenetic changes within various components of the TME, especially CAFs, paving the way for breakthroughs in diminishing lung cancer progression.
